# Neural stem cells as potential mediators of prenatal dietary stress through epigenetic mechanisms

**DOI:** 10.1016/j.stemcr.2026.102996

**Published:** 2026-07-02

**Authors:** Itsuki Kageyama, Hiroya Yamada, Mirai Yamazaki, Takuya Wakasugi, Yuri Kamiya, Masaki Ohshiro, Manaka Ito, Yoshiki Tsuboi, Takashi Watanabe, Genki Mizuno, Yoshitaka Ando, Hiroaki Ishikawa, Koji Suzuki, Koji Ohashi, Eiji Munetsuna

**Affiliations:** 1 Department of Hygiene, Fujita Health University School of Medicine, Toyoake, Japan; 2 Department of Preventive Medical Sciences, Fujita Health University School of Medical Sciences, Toyoake, Japan; 3 Department of Informative Clinical Medicine, Fujita Health University School of Medical Sciences, Toyoake, Japan; 4 Division of Gene Regulation, Oncology Innovation Center, Fujita Health University, Toyoake, Japan; 5 Department of Medical Technology, Tokyo University of Technology School of Health Sciences, Ota, Japan; 6 Department of Animal Science and Biotechnology, Azabu University School of Veterinary Medicine, Sagamihara, Japan

**Keywords:** DOHaD, fructose, osteopontin, DNA methylation, stem cell programming, epigenetics

## Abstract

The developmental origins of health and disease (DOHaD) hypothesis suggests that environmental exposures during development can induce long-term health effects, yet the cellular origin of such persistence remains unclear. Here, we suggest that neural stem cells (NSCs) may develop aberrant properties that persist with possible involvement of epigenetic mechanisms linking maternal dietary stress to neurocognitive impairments. In a rat model of maternal high-fructose corn syrup intake, offspring showed hippocampus-dependent memory deficits and reduced neurogenesis. NSCs from fetal and adolescent hippocampi exhibited persistent dysfunction with transcriptomic dysregulation. Mechanistically, transient downregulation of DNA methyltransferase 3A in fetal NSCs was associated with sustained repression of secreted phosphoprotein 1, encoding intracellular osteopontin (iOPN). iOPN overexpression partially restored NSC function, supporting a potential causal link. Our study proposes a DOHaD framework in which stem cells with lasting alterations may retain epigenetic traces of early life stress, with implications for organ systems and disease risk.

## Highlights

• Maternal dietary stress impairs offspring memory and neurogenesis
• Persistent epigenetic changes are induced in neural stem cells
• Neural stem cell dysfunction persists beyond fetal stages
• This study supports a stem cell-centered framework for developmental programming

## Graphical abstract

**Figure:**
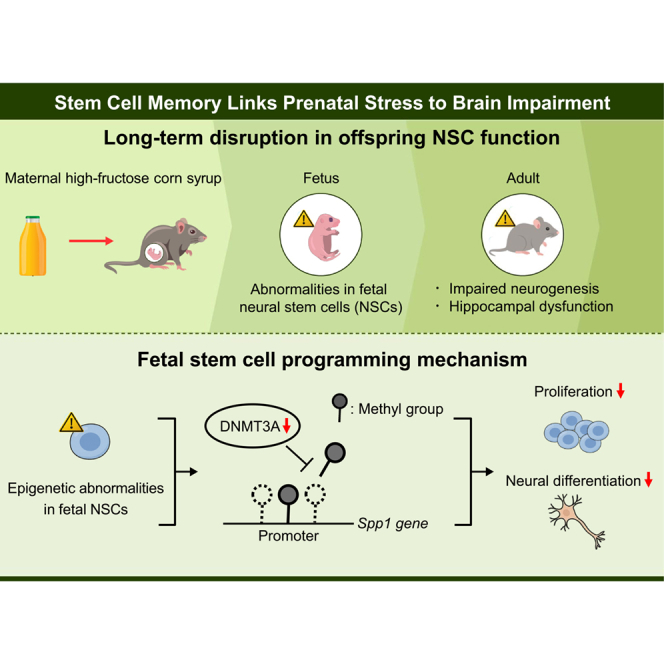

## Teaser

Environmental exposures during development can have lasting health effects, but the cellular origin of this persistence remains unclear. Neural stem cells may retain epigenetic traces of early life stress, supporting a role in developmental programming.

## Introduction

The developmental origins of health and disease (DOHaD) hypothesis posits that environmental conditions during early development can program long-term disease susceptibility (Barker, 1998; Van den Bergh, 2011; Siddeek and Simeoni, 2022). Among these, maternal dietary imbalance—such as excess fructose consumption—is increasingly recognized as a critical contributor to metabolic and neurological dysfunction in offspring (Ando et al., 2022; Munetsuna et al., 2021; Smith et al., 2022; Thompson and DeBosch, 2021; Yamada et al., 2019; Yamazaki et al., 2018). While epidemiological and experimental studies have shown that early life nutritional insults can have lasting effects on brain development and cognition (Freitas-Vilela et al., 2018; Galera et al., 2018; Gould et al., 2018; Roseboom et al., 2011; Yamazaki et al., 2018), the cellular origin and mechanistic underpinnings of such persistent outcomes remain poorly understood.

Recent advances in DOHaD research have implicated epigenetic modifications—including DNA methylation and chromatin remodeling—as key mechanisms linking prenatal environmental exposures to lifelong physiological change (Van den Bergh, 2011; Bianco-Miotto et al., 2017; Siddeek and Simeoni, 2022). However, most studies have focused on differentiated tissues or mature cell types, overlooking the possibility that undifferentiated, self-renewing stem cells might act as the source of long-term programming. We hypothesized that stem cells may retain molecular traces of developmental exposures, which could later influence tissue function and health.

Neural stem cells (NSCs), which are essential for brain development and plasticity (Chen et al., 2025; Jin, 2016; Zhao et al., 2008), offer a compelling model to examine this concept. NSCs retain epigenetic marks, possess lineage potential, and persist into adulthood to support neurogenesis (Li and Guo, 2021; Ma et al., 2010; Podobinska et al., 2017). Thus, even modest epigenetic reprogramming of NSCs by environmental stress might be propagated across developmental stages, potentially contributing to neurodevelopmental and cognitive disorders. Previous studies have shown that early life adversity and maternal dietary challenges can alter hippocampal neurogenesis and cognitive function in offspring (Bilbo and Tsang, 2010; Oomen et al., 2010; Yamazaki et al., 2018). In addition, hippocampal transcriptomic alterations as well as gene-specific epigenetic regulation of individual loci such as brain-derived neurotrophic factor have been reported following such exposures (Roth et al., 2009; Suri et al., 2013; Wei et al., 2012; Yamazaki et al., 2018). However, these studies have largely focused on tissue-level or differentiated neuronal outcomes. Yet the role of NSCs in mediating DOHaD-related outcomes has remained largely unexplored.

In this study, we explored the possibility that maternal high-fructose corn syrup (HFCS) intake produces long-lasting changes in hippocampal NSCs, with epigenetic factors potentially contributing to altered neurogenesis and behavior in offspring. We identified a transient downregulation of the *de novo* DNA methyltransferase (*Dnmt3a*) in fetal NSCs, which is associated with sustained hypomethylation and reduced expression of secreted phosphoprotein 1 (*Spp1*)—encoding intracellular osteopontin (iOPN), a key regulator of NSC function. These epigenetic changes resulted in functional impairments in NSC proliferation and neural differentiation that are already present at the fetal stage and persist into the postnatal period and adulthood. Behaviorally, these molecular and cellular alterations may contribute to hippocampus-dependent cognitive dysfunction in adult offspring. Together, our findings suggest that NSCs can serve as important mediators of environmental programming, providing a framework to understand the cellular basis of DOHaD.

## Results

### Maternal HFCS intake impairs hippocampus-dependent memory and adult neurogenesis in offspring

**Figure 1:**
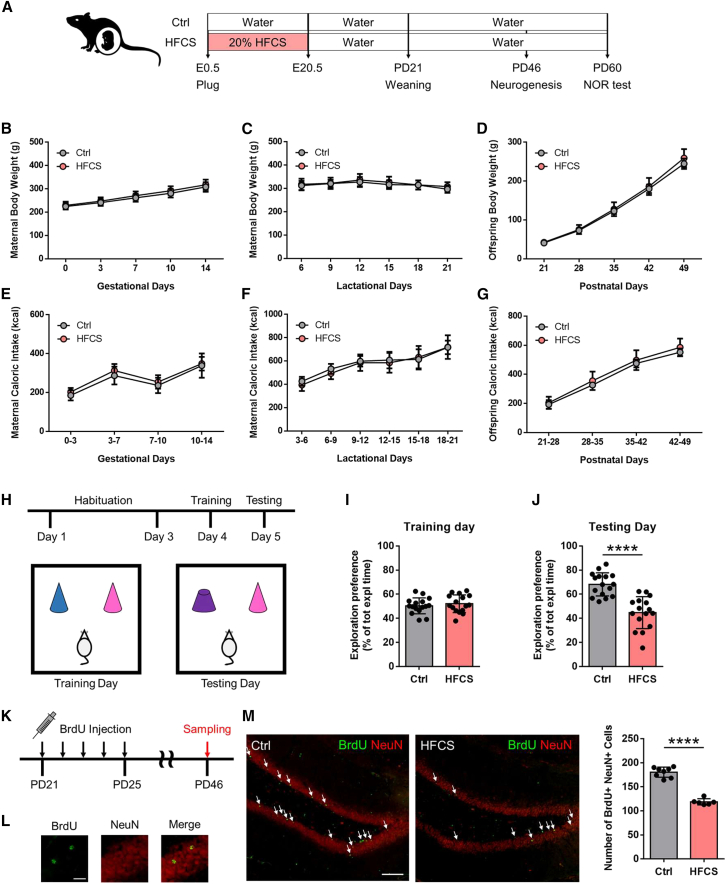
Maternal HFCS intake impairs hippocampus-dependent memory and adult neurogenesis in offspring (A) Animal models and experimental schedule for analysis of hippocampal function in offspring. (B–D) Body weight of dams and offspring during the experimental period (dam: *n* = 13–22/group; offspring: *n* = 16–24/group). (E–G) Caloric intake of dams and offspring during the experimental period. Caloric intake indicates the sum from diets and drinking water (dam: *n* = 13–22/group; offspring: *n* = 7–20/group). (H) Schematic illustrating the assessment of hippocampal-dependent learning and memory. (I and J) The percentage of exploration time spent on the novel object on the training (I) and testing day (J) (dam: *n* = 5–6/group; offspring: *n* = 16/group) in NOR test. (K) Schematic of the experimental protocol of adult neurogenesis analysis in the hippocampal DG. (L) Representative image of immunostaining for BrdU (green) and NeuN (red). Scale bar, 20 μm. (M) Immunostaining for BrdU and NeuN in the hippocampal DG (dam: *n* = 3–4/group; offspring: *n* = 6–8/group). White markers indicate BrdU^+^ NeuN^+^ cells. Scale bar, 100 μm. Ctrl, control group; HFCS, HFCS group. *n* = number of animals analyzed. Values are presented as means ± SD. For (B*–*G), statistical analysis was performed by one-way ANOVA. For (I, J, and M), statistical analysis was performed by Student’s *t* test. Nonsignificant comparisons are not shown. ^∗∗∗∗^*p* < 0.0001.

Dams were fed with normal water or 20% HFCS solution immediately after pregnancy (Figure 1A). Maternal HFCS intake had no effect on the body weight and caloric intake of either dams or offspring among the groups during the experimental period (Figures 1B–1G). In addition, a pair-feeding study that was conducted with 20% glucose solution also revealed no significant difference in the body weight or caloric intake (Figures S1A–S1G). To examine whether maternal HFCS exposure affects cognitive function in offspring, we subjected adult rats to the novel object recognition (NOR) test (Figures 1H–1J). Offspring from HFCS-fed dams showed significantly reduced recognition memory compared with control, as evidenced by decreased exploration preference during the testing phase (Figure 1J; *p* < 0.0001, Cohen’s d = 2.02, 95% confidence interval [CI], 1.15–2.87). To investigate the underlying neurobiological basis, we examined hippocampal neurogenesis by bromodeoxyuridine (BrdU) incorporation and immunohistochemical staining for NeuN. HFCS offspring exhibited a significant reduction in the number of BrdU^+^ NeuN^+^ cells in the dentate gyrus (DG) of the hippocampus (Figures 1K–1M), suggesting impaired adult neurogenesis (Figure 1M; *p* < 0.0001, Cohen’s d = 6.84, 95% CI, 3.92–9.72). No significant effects on cognitive behavior or neurogenesis were observed in the glucose group (Figures S1H–S1J).

### Persistent NSC dysfunction induced by maternal HFCS exposure

**Figure 2:**
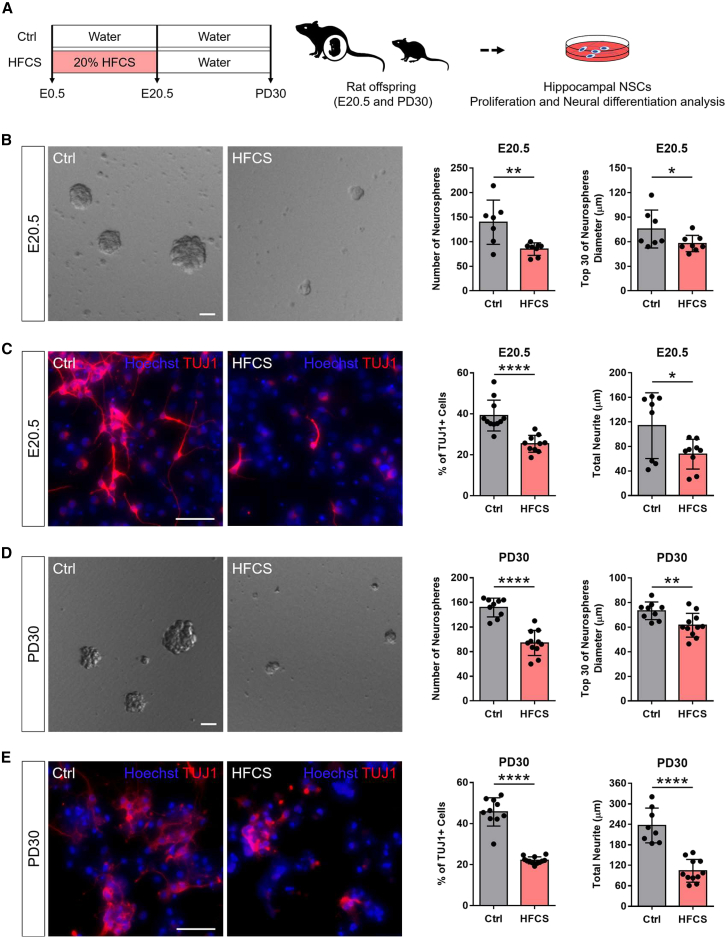
Persistent NSC dysfunction induced by maternal HFCS exposure (A) Schematic of isolation and culture of NSCs from rat offspring in HFCS models. (B) Neurosphere formation and quantification of neurosphere diameters at E20.5 (dam: *n* = 5/group; offspring: *n* = 7–8/group). Scale bar, 50 μm. (C) Immunostaining for quantification of the percentage and total neurite length of TUJ1^+^ cells differentiated from hippocampal NSCs at E20.5 (dam: *n* = 4–6/group; offspring: *n* = 8–11/group). Scale bar, 50 μm. (D) Neurosphere formation and quantification of neurosphere diameters at PD30 (dam: *n* = 4/group; offspring: *n* = 9–11/group). Scale bar, 50 μm. (E) Immunostaining for quantification of the percentage and neurite length of TUJ1^+^ cells differentiated from hippocampal NSCs at PD30 (dam: *n* = 4–5/group; offspring: *n* = 8–12/group). Scale bar, 50 μm. Ctrl, control group; HFCS, HFCS group. *n* = number of animals analyzed. Values are presented as means ± SD. All statistical analyses were performed by Student’s *t* test. Nonsignificant comparisons are not shown. ^∗^*p* < 0.05, ^∗∗^*p* < 0.01, ^∗∗∗∗^*p* < 0.0001.

We next evaluated NSC function by isolating hippocampal NSCs from fetal (embryonic day [E] 20.5) and adolescent (postnatal day [PD]30) offspring (Figure 2A). Neurosphere assays revealed that NSCs from HFCS offspring formed fewer and smaller neurospheres across developmental stages compared with controls (Figures 2B–2D). Quantification showed reduced neurosphere number and diameter at E20.5 (number: *p* < 0.01, Cohen’s d = 1.71, 95% CI, 0.48–2.89; diameter: *p* < 0.05, Cohen’s d = 1.02, 95% CI, −0.08–2.09) and PD30 (number: *p* < 0.0001, Cohen’s d = 3.15, 95% CI, 1.78–4.48; diameter: *p* < 0.01, Cohen’s d = 1.36, 95% CI, 0.36–2.33). Immunocytochemistry further demonstrated decreased rates of neuronal differentiation and shortened neurite length in NSCs from HFCS-exposed offspring (Figures 2C–2E), indicating persistent deficits in NSC proliferation and neural differentiation. The differentiation rate and total neurite length were decreased at E20.5 (differentiation: *p* < 0.0001, Cohen’s d = 2.25, 95% CI, 1.12–3.34; neurite length: *p* < 0.05, Cohen’s d = 1.14, 95% CI, 0.09–2.16) and PD30 (differentiation: *p* < 0.0001, Cohen’s d = 4.93, 95% CI, 3.18–6.64; neurite length: *p* < 0.0001, Cohen’s d = 3.20, 95% CI, 1.78–4.58). No such effects were observed in the glucose group (Figure S3).

### Transcriptomic dysregulation in fetal NSCs following HFCS exposure at E20.5

**Figure 3:**
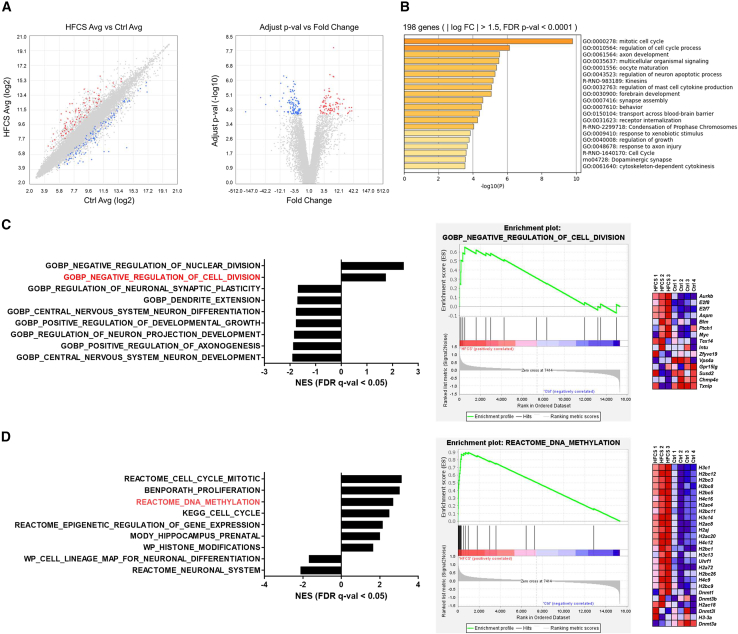
Transcriptomic dysregulation in fetal NSCs following HFCS exposure at E20.5 (A) Scatterplot (left) and volcano plot (right) of differentially expressed genes (DEGs) from the hippocampal NSCs at E20.5 (dam: *n* = 2/group; offspring: *n* = 3–4/group). Red and blue dots indicate statistical DEGs. (B) Gene Ontology (GO) analysis of DEGs in hippocampal NSCs at E20.5 (dam: *n* = 2/group; offspring: *n* = 3–4/group). (C and D) Gene set enrichment analysis (GSEA) results of hippocampal NSCs at E20.5. The heatmaps display all genes belonging to the indicated enriched gene sets highlighted in red (dam: *n* = 2/group; offspring: *n* = 3–4/group). Ctrl, control group; HFCS, HFCS group. *n* = number of animals analyzed.

Transcriptome profiling of hippocampal NSCs at E20.5 identified 198 differentially expressed genes (DEGs; false discovery rate <0.05), including 111 upregulated and 87 downregulated genes in HFCS-exposed offspring (Figure 3A). Among the up- or down-regulated genes, a list of genes with high expression variability is provided in the supporting information (Tables S1 and S2). Gene Ontology analysis revealed enrichment of DEGs related to cell division and neuronal development (Figure 3B). Gene set enrichment analysis further showed upregulation of pathways associated with negative regulation of cell division and downregulation of axonogenesis and neurogenesis-related gene sets (Figures 3C and 3D). Notably, pathways involved in DNA methylation and epigenetic gene regulation were also dysregulated.

### Maternal HFCS intake suppresses *Dnmt3a* and downregulates *Spp1* in NSCs

**Figure 4:**
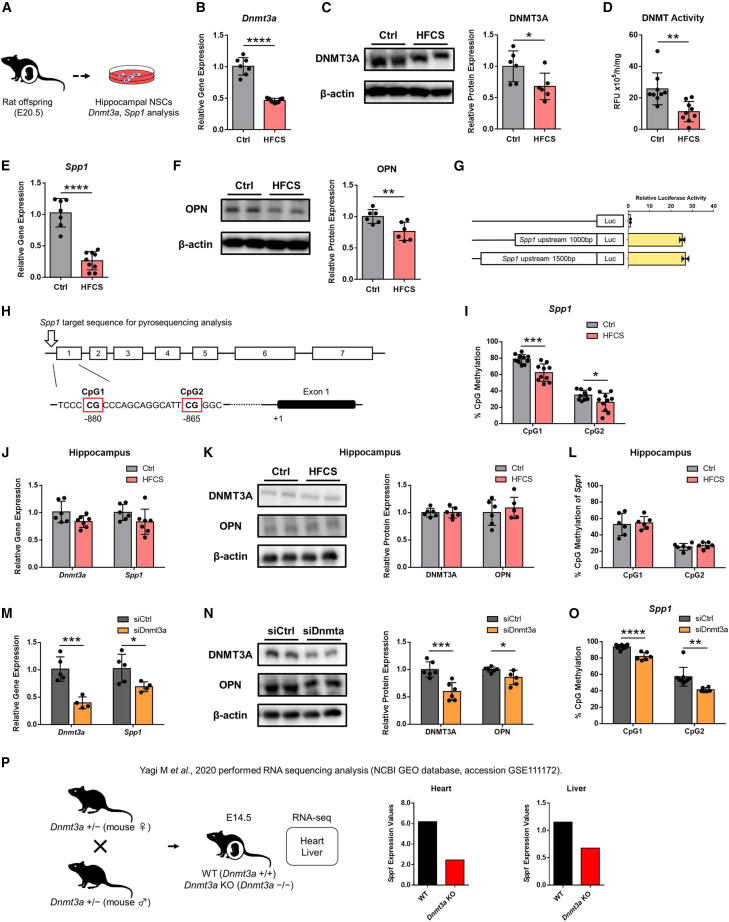
Maternal HFCS intake suppresses *Dnmt3a* and downregulates *Spp1* in NSCs (A) Primary culture of hippocampal NSCs at E20.5. (B and C) qPCR (B) and western blot (WB) (C) analysis showed *Dnmt3a* expression of hippocampal NSCs at E20.5 (dam: *n* = 2–3/group; offspring: *n* = 6–9/group). (D) DNMT activity analysis of hippocampal NSCs at E20.5 (dam: *n* = 9/group; offspring: *n* = 27/group, pooled as 3 offspring per dam). (E and F) qPCR (E) and WB (F) analysis showed *Spp1* (OPN) expression of hippocampal NSCs at E20.5 (dam: *n* = 2–3/group; offspring: *n* = 6–9/group). (G) Luciferase assay to assess promoter activity upstream (−1,000 bp, −1,500 bp) of the *Spp1* gene in rat hippocampal NSCs (*n* = 2/group). (H) The analyzed sequence of the *Spp1* promoter region is shown schematically. (I) DNA methylation analysis of *Spp1* gene by pyrosequencing of hippocampal NSCs at E20.5 (dam: *n* = 4–5/group; offspring: *n* = 10/group). (J and K) Hippocampal tissue qPCR (J) and WB (K) analysis of *Dnmt3a* and *Spp1* (OPN) at E20.5 (dam: *n* = 2–3/group; offspring: *n* = 6–9/group). (L) DNA methylation analysis of *Spp1* gene by pyrosequencing of hippocampus at E20.5 (dam: *n* = 3/group; offspring: *n* = 6/group). (M and N) qPCR (M) and WB (N) analysis of *Dnmt3a*-knockdown rat hippocampal NSCs (*n* = 4–6/group). (O) DNA methylation analysis of *Spp1* gene by pyrosequencing of *Dnmt3a*-knockdown rat hippocampal NSCs (*n* = 6–9/group). (P) Experimental overview of RNA sequencing performed by Yagi M et al. *Spp1* gene expression in heart and liver of *Dnmt3a*-knockout (KO) mice generated from open database (NCBI GEO database, accession GSE111172). Ctrl, control group; HFCS, HFCS group. siCtrl, siDnmt3a control group; siDnmt3a, siDnmt3a group. (B–F) and (I–L), *n* = number of animals analyzed. (G) and (M–O), *n* = number of independent experiments. Values are presented as means ± SD. All statistical analyses were performed by Student’s *t* test. Nonsignificant comparisons are not shown. ^∗^*p* < 0.05, ^∗∗^*p* < 0.01, ^∗∗∗^*p* < 0.001, ^∗∗∗∗^*p* < 0.0001.

Among genes included in the enriched DNA methylation-related gene set identified by GSEA, *Dnmt3a* emerged as the most significantly downregulated gene (Figure 3D; Table S3). qPCR, western blotting, and enzymatic assays confirmed decreased *Dnmt3a* mRNA, protein, and DNMT activity in fetal NSCs from HFCS offspring (Figures 4A–4D). Full-length, uncropped western blot images are provided (Figure S7). We investigated the expression of 12 genes based on DEGs that were suggested to be associated with NSC function (Figure 4E; Figure S4). In particular, *Spp1*, a gene involved in NSC maintenance, was downregulated at E20.5 (Figures 4E and 4F). Methylation analysis of the *Spp1* promoter showed reduced CpG methylation at key regulatory regions unlike other 11 genes (Figures 4G–4I; Figure S4). Hippocampal expression of *Dnmt3a* and *Spp1* was not altered between the two groups (gene and protein expression: Figures 4J and 4K respectively). The DNA methylation level of *Spp1* was also unchanged (Figure 4L). *Dnmt3a* knockdown in NSCs using small interfering RNA (siRNA) recapitulated the reduction in *Spp1* expression and promoter hypomethylation (Figures 4M–4O), confirming that *Dnmt3a* directly regulates *Spp1* epigenetically. These findings suggest that transient suppression of *Dnmt3a* during fetal development programs long-term *Spp1* repression in NSCs. Supporting this finding, Yagi M et al. reported that *Dnmt3a* knockout reduced *Spp1* expression in the heart and liver (Figure 4P) (Yagi et al., 2020). Collectively, reduced expression of *Dnmt3a* may lead to DNA hypomethylation of the *Spp1* promoter, leading to decreased *Spp1* expression.

### Persistent repression of *Spp1* in postnatal NSCs

**Figure 5:**
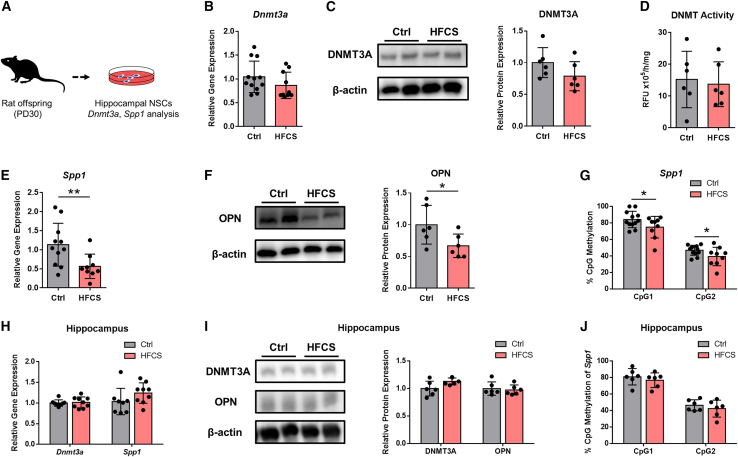
Persistent repression of *Spp1* in postnatal NSCs (A) Primary culture of hippocampal NSCs at PD30. (B and C) qPCR (B) and WB (C) analysis showed *Dnmt3a* expression of hippocampal NSCs at PD30 (dam: *n* = 3–4/group; offspring: *n* = 6–12/group). (D) DNMT activity analysis of hippocampal NSCs at PD30 (dam: *n* = 6/group; offspring: *n* = 18/group, pooled as 3 offspring per dam). (E and F) qPCR (E) and WB (F) analysis showed *Spp1* (OPN) expression of hippocampal NSCs at PD30 (dam: *n* = 3–4/group; offspring: *n* = 6–11/group). (G) DNA methylation analysis of *Spp1* gene by pyrosequencing of hippocampal NSCs at PD30 (dam: *n* = 4/group; offspring: *n* = 9–12/group). (H and I) Hippocampal tissue qPCR (H) and WB (I) analysis of *Dnmt3a* and *Spp1* (OPN) in PD30 (dam: *n* = 3–5/group; offspring: *n* = 6–9/group). (J) DNA methylation analysis of *Spp1* gene by pyrosequencing of hippocampus at PD30 (dam: *n* = 3/group; offspring: *n* = 6/group). Ctrl, control group; HFCS, HFCS group. *n* = number of animals analyzed. Values are presented as means ± SD. All statistical analyses were performed by Student’s *t* test. Nonsignificant comparisons are not shown. ^∗^*p* < 0.05, ^∗∗^*p* < 0.01.

At PD30, *Dnmt3a* expression and activity had normalized in HFCS offspring (Figures 5A–5D), yet *Spp1* expression remained reduced unlike other 11 genes and the *Spp1* promoter remained hypomethylated (Figures 5E–5G; Figure S5), indicating a sustained epigenetic imprint established during fetal life. At several other CpG sites as well, hypomethylation persisted from E20.5 to PD30 (Figure S6). Analysis of hippocampal tissue at PD30 showed no significant differences between the two groups (Figures 5H–5J). These data indicate that the transient decrease in DNMT activity at E20.5 leads to the hypomethylation of *Spp1*, which persists until PD30.

### Functional relevance of *Spp1* in NSCs

**Figure 6:**
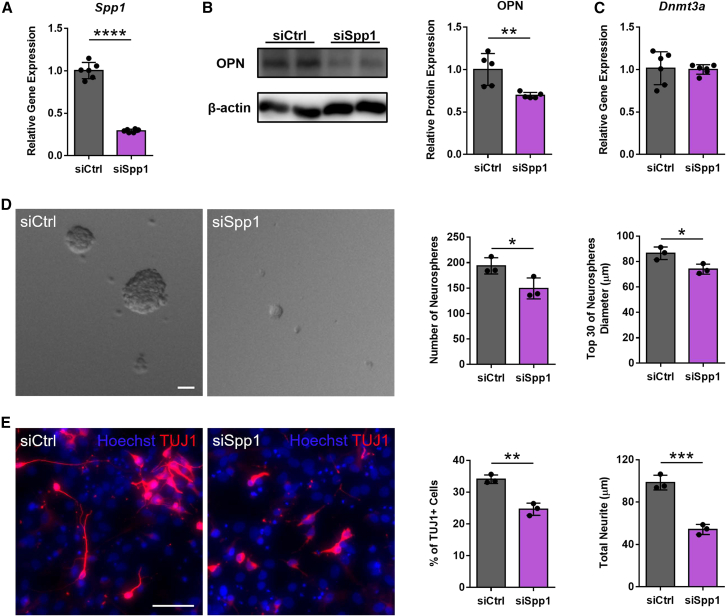
Functional relevance of *Spp1* in NSCs (A and B) qPCR (A) and WB (B) analyses of *Spp1* knockdown hippocampal NSCs (*n* = 5–6/group). (C) qPCR analysis showed *Dnmt3a* expression of *Spp1*-knockdown hippocampal NSCs (*n* = 6/group). (D) Neurosphere formation and quantification of neurosphere diameters of *Spp1*-knockdown hippocampal NSCs (*n* = 3/group). Scale bar, 50 μm. (E) Immunostaining for quantification of the percentage and total neurite length of TUJ1^+^ cells differentiated from *Spp1*-knockdown hippocampal NSCs (*n* = 3/group). Scale bar, 50 μm. siCtrl, siSpp1 control group; siSpp1, siSpp1 group. *n* = number of independent experiments. Values are presented as means ± SD. All statistical analyses were performed by Student’s *t* test. Nonsignificant comparisons are not shown. ^∗^*p* < 0.05, ^∗∗^*p* < 0.01, ^∗∗∗^*p* < 0.001, ^∗∗∗∗^*p* < 0.0001.

siRNA-mediated knockdown of *Spp1* in NSCs reduced neurosphere size and number, impaired neural differentiation, and shortened neurite length (Figure 6), confirming that *Spp1* is essential for NSC function.

### iOPN restores NSC function impaired by maternal HFCS intake

**Figure 7:**
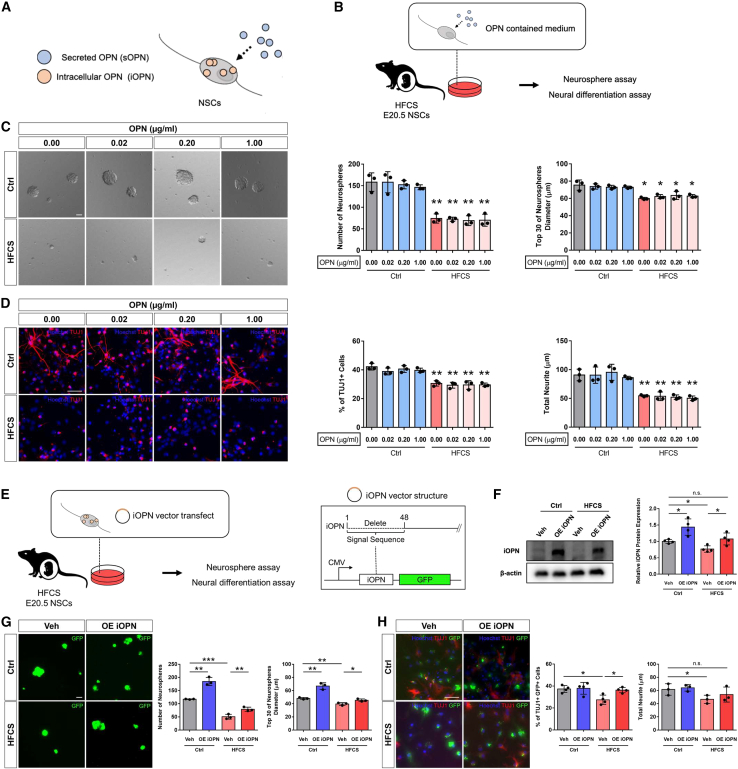
iOPN restores NSC function impaired by maternal HFCS intake (A) Schematic of the effects of two types of OPN (secreted OPN, sOPN; intracellular OPN, iOPN). (B) Schematic of an experiment to identify whether sOPN recovers hippocampal NSC function from HFCS rats. (C) Neurosphere formation and quantification of neurosphere diameters of sOPN-like treated hippocampal NSCs (*n* = 3/group). Scale bar, 50 μm. Statistical analysis was shown vs. Ctrl 0.00. (D) Immunostaining for quantification of the percentage and total neurite length of TUJ1^+^ cells differentiated from sOPN-like treated hippocampal NSCs (*n* = 3/group). Scale bar, 50 μm. Statistical analysis was shown vs. Ctrl 0.00. (E) Schematic of an experiment to identify whether iOPN recovers hippocampal NSC function from HFCS rats. Brief structural schematic of the iOPN vector. (F) WB analysis of iOPN in vector-transfected hippocampal NSCs (*n* = 3/group). (G) Neurosphere formation and quantification of neurosphere diameters of hippocampal NSCs overexpressing iOPN (*n* = 3/group). Scale bar, 50 μm. (H) Immunostaining for quantification of the percentage and total neurite length of TUJ1^+^ GFP^+^ cells differentiated from hippocampal NSCs overexpressing iOPN (*n* = 3–4/group). Scale bar, 50 μm. Ctrl, control group; HFCS, HFCS group; Veh, vehicle group; OE iOPN, over expression iOPN group. *n* = number of independent experiments. Values are presented as means ± SD. All statistical analyses were performed by Student’s *t* test with Holm-Bonferroni correction for multiple comparisons. Nonsignificant comparisons are not shown. ^∗^*p* < 0.05, ^∗∗^*p* < 0.01, ^∗∗∗^*p* < 0.001; ns, not significant.

To determine whether restoring *Spp1* function could rescue HFCS-induced NSCs deficits, we overexpressed iOPN in NSCs using a signal peptide-deleted construct. Although secreted osteopontin (sOPN) treatment had no effect (Figures 7A–7D), iOPN overexpression rescued NSC proliferation and differentiation in HFCS offspring (Figures 7E–7H). Quantitative analyses confirmed the restoration of neurosphere formation and neuronal differentiation in HFCS-derived NSCs (Figures 7G and 7H; sphere number: *p* < 0.01, Cohen’s d = 3.28, 95% CI, 0.52–5.93; differentiation rate: *p* < 0.05, Cohen’s d = 2.54, 95% CI, 0.52–4.47). These results establish iOPN as the key effector downstream of *Spp1* in maintaining NSC function after environmental stress. Together, these findings demonstrate that maternal HFCS intake epigenetically programs NSCs by transiently suppressing *Dnmt3a*, resulting in persistent repression of *Spp1* and long-term deficits in neurogenesis and cognition.

## Discussion

Our study suggests that NSCs may develop aberrant properties that persist as long-term traces of maternal dietary stress, with possible involvement of epigenetic mechanisms. Specifically, we observed that transient suppression of *Dnmt3a* in fetal hippocampal NSCs was associated with sustained repression of *Spp1* via promoter hypomethylation, which may contribute to impaired NSC function and cognitive deficits that may extend into adulthood. These findings suggest that NSCs could encode and transmit the effects of prenatal environmental exposure, supporting a potential framework for stem cell-centered developmental programming within the DOHaD paradigm.

Previous DOHaD studies have primarily focused on tissue-level DNA methylation analysis to investigate how prenatal environmental factors shape long-term health risks (Ando et al., 2022; Burdge et al., 2007; Lillycrop et al., 2008; Munetsuna et al., 2021; Smith et al., 2022; Somm et al., 2009; Thompson and DeBosch, 2021; Yamada et al., 2019; Yamazaki et al., 2018; Yuan et al., 2018). However, this approach has fundamental limitations. Many tissues undergo continuous turnover, where differentiated cells are gradually replaced by new ones over time (Banjac et al., 2023; Cho et al., 2019; O’Brien, 2022). Therefore, it seems unlikely that DNA methylation abnormalities acquired at fetal stage are consistently maintained throughout life. Moreover, because tissue-level methylation analysis primarily reflects the epigenetic state of differentiated cells, it is difficult to determine how fetal epigenetic changes persist over time and contribute to long-term effects. However, our study supports the possibility that the epigenetic memory of DOHaD may be retained in stem cells. Specifically, we observed that maternal HFCS exposure induces persistent DNA methylation abnormalities in hippocampal NSCs, which may contribute to long-term neurodevelopmental impairments. It is considered that, because NSCs possess self-renewing capacity unlike differentiated cells, NSCs may have the ability to maintain and transmit epigenetic alterations through life.

NSCs can be broadly classified into two distinct populations: those that are actively involved in tissue formation during development and those that persist throughout life to maintain tissue homeostasis (Llorente et al., 2022; de Morree and Rando, 2023; Obernier and Alvarez-Buylla, 2019; Urbán and Guillemot, 2014). Our findings suggest that both populations may be affected by maternal HFCS exposure. Disruptions in the developmental NSC pool could lead to abnormalities in early neurogenesis and brain structure formation, while impairments in the lifelong NSC pool may compromise adult neurogenesis, contributing to long-term cognitive decline. Given that NSCs play a critical role in both neurodevelopment and neural maintenance, our results suggest that prenatal HFCS exposure may have lasting consequences on brain function by dysregulating both developmental and lifelong NSC populations.

The reduction in adult DG neurogenesis observed in this study is consistent with the involvement of NSCs. Although this readout reflects the combined outcome of multiple sequential processes and may also be influenced by niche-derived signals, our findings demonstrating impaired NSC proliferation and neuronal differentiation support a primary contribution of intrinsic NSC dysfunction. Notably, a previous maternal HFCS exposure study reported no persistent increase in hippocampal pro-inflammatory cytokine expression at PD21 or PD60 (Yamazaki et al., 2023), and increased apoptosis was not detected in a related fructose exposure model (Yamazaki et al., 2018). These findings suggest that the reduced neurogenesis observed here is unlikely to be solely explained by chronic neuroinflammation or enhanced cell death. Taken together, our data support a model in which intrinsic alterations in NSCs are a major contributor to the observed phenotype.

A key molecular insight from this study is evidence for a potential *Dnmt3a-Spp1* regulatory axis as a mediator of this epigenetic memory. *Dnmt3A* is a *de novo* DNA methyltransferase that plays a pivotal role in the establishment of epigenomic patterns during development (Chen and Zhang, 2020; Okano et al., 1999). Although *Dnmt3a* expression was significantly reduced at E20.5, it had returned to baseline levels by PD30. Nevertheless, the hypomethylation and reduced expression of *Spp1* persisted at PD30, suggesting that a temporary disruption of epigenetic regulation during development can result in sustained functional abnormalities—a phenomenon suggestive of epigenetic memory. Importantly, we identified the intracellular isoform of osteopontin (iOPN), encoded by *Spp1*, as essential for NSC maintenance. Functional impairment induced by maternal HFCS exposure or *Spp1* knockdown was rescued by iOPN overexpression, but not by extracellular sOPN, underscoring the specificity of intracellular mechanisms. These results support the possibility that transient epigenetic disruptions during development may lead to long-lasting functional consequences in stem cell populations, not only by affecting canonical stem cell regulators but also by altering non-traditional but functionally critical genes. This expands the conceptual framework of DOHaD by positioning stem cells—and their epigenetic memory—as key mediators of long-term effects of early life environmental exposures. These insights may extend beyond the nervous system. Other long-lived stem cell populations, such as hematopoietic or mesenchymal stem cells, might similarly retain epigenetic memory of prenatal exposures. This broader framework may help explain how environmental stressors during gestation exert multisystemic and lifelong effects, from metabolic disease to immune dysfunction. Targeting such epigenetic memory in stem cells could become a novel strategy for mitigating DOHaD-related health outcomes.

Although promoter CpG methylation is often associated with transcriptional repression, this relationship is context dependent (de Mendoza et al., 2022). In the present study, reduced *Dnmt3a* expression was associated with hypomethylation of the *Spp1* promoter accompanied by decreased *Spp1* expression. This counterintuitive observation suggests that *Dnmt3a* may not regulate *Spp1* transcription through a simple linear promoter methylation-dependent mechanism. One possible explanation is that loss of methylation facilitates the binding of methylation-sensitive repressors, thereby attenuating transcription. Alternatively, *Dnmt3a* downregulation may influence broader chromatin regulatory mechanisms. These findings support a context-dependent and potentially indirect epigenetic regulatory mechanism underlying the *Dnmt3a*-*Spp1* relationship.

From an evolutionary perspective, retaining environmental information specifically in long-lived, self-renewing stem cells may offer a strategic advantage by allowing the organism to anticipate postnatal environments and stably adjust developmental and physiological trajectories over time. Within the framework of the DOHaD hypothesis, it may not be merely transient cellular responses, but epigenetic memory embedded in stem cells that may serve as a foundational mechanism to imprint prenatal environmental conditions into the long-term functional settings of metabolic, endocrine, and immune systems, thereby enhancing survival and reproductive success.

However, when postnatal environments deviate from prenatal expectations, such stem cell-encoded memories may become maladaptive, increasing susceptibility to disease and highlighting the evolutionary trade-off between adaptation and plasticity.

Thus, the retention of environmental information in stem cells—cells that not only persist throughout life but also continually regenerate tissues—may represent a fundamental mechanism through which early life experiences exert lasting effects on health and disease risk.

This study has several limitations. While we identified a transient reduction in DNMT activity in NSCs and focused on *Spp1* as a key epigenetically regulated gene, it is possible that other functionally important genes are also epigenetically altered. Further comprehensive epigenomic analyses, such as whole-genome bisulfite sequencing or chromatin immunoprecipitation sequencing, are warranted to explore additional targets. In addition, future cell type-specific and niche-focused analyses *in vivo* will be required to more precisely delineate intrinsic and extrinsic mechanisms underlying the observed phenotype. Moreover, as this study was conducted using an animal model, future epidemiological research will be essential to evaluate the translational relevance of these findings to human health.

In conclusion, our results suggest that maternal HFCS consumption during pregnancy may induce lasting alterations in NSCs, potentially involving epigenetic mechanisms and contributing to long-term impairments in neurogenesis and cognition. These findings suggest that stem cells could act as mediators within the DOHaD framework and raise the possibility that interventions aimed at epigenetic regulation in stem cells may help mitigate the long-term consequences of early life environmental stressors.

## Resource availability

### Lead contact

Requests for further information, resources, and reagents should be directed to and will be fulfilled by the lead contact, Hiroya Yamada (hyamada@fujita-hu.ac.jp).

### Materials availability

This study did not generate new unique reagents.

### Data and code availability

All data reported in this paper will be shared by the lead contact upon request. This study did not generate any unique code. The RNA-seq data analyzed in this paper were obtained from GEO under accession number GSE111172 (Yagi et al., 2020). The microarray data generated in this study have been deposited in GEO under accession number GSE332845. Any additional information required to reanalyze the data reported in this paper is available from the lead contact upon request.

## Acknowledgments

This study was supported by Grants-in-Aid for Scientific Research (B) (grant numbers 20H04134 and 24K02692), Scientific Research (C) (grant numbers 22K10494), and Young Scientists (grant number 23K13917) from the 10.13039/501100001691Japan Society for the Promotion of Science (JSPS), funded by the 10.13039/501100001700Ministry of Education, Culture, Sports, Science and Technology (MEXT) of Japan.

## Author contributions

Conceptualization, H.Y. and E.M.; methodology, M.Y., Y.T., G.M., Y.A., H.I., K.S., and K.O.; investigation, I.K., T. Wakasugi, Y.K., M.O., and M.I.; writing – original draft, I.K., H.Y., and E.M.; writing – review and editing, H.Y. and E.M.; funding acquisition, Y.A., H.Y., and E.M.; resources, T. Watanabe; supervision, H.Y. and E.M.

## Declaration of interests

The authors declare no competing interests.

## STAR★Methods

### Key resources table

**Table:** 

REAGENT or RESOURCE	SOURCE	IDENTIFIER
**Antibodies**		

Rabbit anti-NeuN	Abcam	Cat # ab128886; RRID: AB_2744676
Rabbit anti-βIII Tubulin (TUJ1)	Abcam	Cat # ab18207; RRID: AB_444319
Rabbit anti-SOX2	Abcam	Cat # ab97959; RRID: AB_2341193
Rabbit anti-GFAP	Abcam	Cat # ab7260; RRID: AB_305808
Rabbit anti-CD11b	Abcam	Cat # ab75476; RRID: AB_1310048
Mouse anti-NESTIN	Abcam	Cat # ab6142; RRID: AB_305313
HRP-Mouse anti-β-actin	Abcam	Cat # ab49900; RRID: AB_867494
HRP-Goat anti-mouse	Abcam	Cat # ab97023; RRID: AB_10679675
HRP-Goat anti-rabbit	Cell Signaling Technology	Cat # 7074; RRID: AB_2099233
Rabbit anti-DNMT3A	Cell Signaling Technology	Cat # 3598; RRID: AB_2277449
Alexa Fluor 488-goat anti-mouse	Thermo Fisher Scientific	Cat # A-11017; RRID: AB_2534084
Alexa Fluor 594-goat anti-rabbit	Thermo Fisher Scientific	Cat # A-11072; RRID: AB_2534116
Mouse anti-BrdU	Millipore	Cat # MAB4072; RRID: AB_95024
Mouse anti-OPN	Santa Cruz Biotechnology	Cat # sc-21742; RRID: AB_2194997

**Chemicals, peptides, and recombinant proteins**		

5-Bromo-2′-deoxyuridine (BrdU)	Sigma-Aldrich	Cat #B5002
Recombinant Rat Osteopontin	R&D Systems	Cat # 6359-OP

**Deposited data**		

Microarray data	This paper	GSE332845
RNA-seq data	(Yagi et al., 2020)	GSE111172

**Experimental models: Organisms/strains**		

Sprague-Dawley rat	Japan SLC, Inc.	N/A
The primary rat hippocampal neural stem cells (NSCs)	This paper	N/A

**Oligonucleotides**		

See Tables S4 and S5 for primer sequences	N/A	N/A

**Software and algorithms**		

ImageJ	National Institutes of Health	https://imagej.nih.gov/ij
Fiji	National Institutes of Health	https://fiji.sc/
Metascape	(Zhou et al., 2019)	https://www.metascape.org
Gene Set Enrichment Analysis	(Subramanian et al., 2005)	https://www.gsea-msigdb.org/gsea/index.jsp
PyroMark Assay Design 2.0	Qiagen	N/A
PyroMark Q24 Advanced	Qiagen	N/A
JMP	SAS	https://www.jmp.com/en_us/home.html
R	R Consortium	http://www.R-project.org
RStudio	RStudio	https://posit.co

### Experimental model and study participant details

#### Animals

The study protocol was approved by the Fujita Health University Animal Ethics Committee. We established an animal model of maternal high fructose consumption as previously described (Ando et al., 2022, 2024). All animals were housed in an environmentally controlled cage at room temperature (23 ± 3°C) under a 12:12 h light-dark cycle. 8-week-old male and female Sprague-Dawley rats (Japan SLC, Hamamatsu, Japan) were acclimatized for 1 week, after which one male rat was housed with one female rat. Gestation was confirmed based on the presence of a vaginal plug, after which female rats were allocated to 2 experimental groups: one group receiving distilled water, one group receiving 20% HFCS solution. All animals had *ad libitum* access to their respective water and standard chow (MF; Oriental Yeast, Tokyo, Japan). 20% HFCS solution was prepared using 75% HFCS (Japan Corn Starch, Tokyo, Japan) and distilled water. HFCS solution was administered throughout gestation from vaginal plug identification until gestational day 20.5, after which it was replaced with distilled water for the remainder of gestation. In addition, paired feeding experiments using a 20% glucose solution were conducted. This group was used exclusively for supplemental experiments (Figures S1–S3). The glucose solution was prepared using D-(+)-glucose dissolved in distilled water at a final concentration of 20%. Rats in the glucose group were provided *ad libitum* access to the glucose solution and standard chow under the same housing conditions as described above.

### Method details

#### Behavior, novel-object recognition assay

Novel object recognition (NOR) tests were performed as previously described (Yamazaki et al., 2018). Rats were subjected to a training session followed by a testing session 24 h later, in which one familiar object was replaced with a novel object. Exploration time for each object was recorded, and recognition memory was expressed as the percentage of time spent exploring the novel object relative to the total exploration time.

#### Immunohistochemistry and quantification of BrdU-labeled cells

To label proliferating cells, rats received intraperitoneal injections of BrdU (100 mg/kg; Sigma-Aldrich, St. Louis, MO, USA) twice daily at 8-h intervals for 5 consecutive days (postnatal days 21–25). Animals were euthanized 3 weeks after the final injection. Brains were collected, fixed in 4% paraformaldehyde, cryoprotected in sucrose, and sectioned coronally at 40 μm. Every eighth section (320 μm apart) throughout the dentate gyrus was processed for BrdU and NeuN double immunofluorescence staining to identify newly generated neurons, as previously described (Tozuka et al., 2005). BrdU^+^ NeuN^+^ cells were quantified in the subgranular zone and granule cell layer of the dentate gyrus, and counts were pooled across both upper and lower blades. Six sections per animal were analyzed at ×20 magnification using a BZ-X800 microscope (Keyence). BrdU^+^ NeuN^+^ cells were considered newly generated neurons that survived and matured after BrdU labeling.

#### Isolation and culture of primary hippocampal NSCs

Hippocampi were removed from male rat offspring (E20.5 and PD30) and dissociated using Neural Tissue Dissociation Kit P (Miltenyi Biotec, Bergisch Gladbach, Germany) according to the manufacturer’s instructions. In PD30 hippocampi, density gradient centrifugation using Percoll (Sigma-Aldrich) was performed to remove non-cellular components. After the last centrifuge, the pellet was suspended in KBM neural stem cell medium (Kohjin Bio, Saitama, Japan) supplemented with EGF and bFGF (Kohjin Bio) and seeded into culture plates. NSCs were grown as neurospheres in ultra-low attachment plates (Corning, NY, USA) or plated on poly-L-ornithine/laminin-coated plastic plates. The medium was changed every 2-3 days, and cells were cultured for 7 days. NSC-enriched populations were obtained by culturing for 7 days in undifferentiated maintenance medium (Figure S2). This culture condition was applied prior to all experiments evaluating NSCs.

#### Proliferation and neural differentiation analysis

Proliferation of NSCs was assessed by neurosphere assay. NSCs were seeded in 96-well plates at a density of 2.0 × 10^3^ cells/well and cultured under floating conditions in neural stem cell maintenance medium supplemented with EGF and bFGF for 7 days. Neurosphere formation was evaluated by measuring sphere diameter, and spheres larger than 30 μm were included in the analysis. For morphometric and differentiation assays, NSCs were seeded on poly-L-ornithine/laminin-coated 96-well plates at a density of 4.0 × 10^4^ cells/well. After preculture, cells were switched to neural induction medium and differentiated for 9 days, with medium replacement every 3 days. Differentiated neurons were identified by immunostaining for βIII-tubulin (TUJ1), and the percentage of TUJ1^+^ cells was calculated based on nuclear staining. Morphological evaluation of neurons was performed using the ImageJ plugin NeuronJ (version 1.4.3; NIH) with reference to other reports (Griffin et al., 2020; Ho et al., 2011). Images acquired with a ×20 objective lens were used to semi-automatically trace neurites and calculate the total neurite length per cell. The mean total neurite length per cell was calculated for each sample and used for comparison between groups.

#### Immunostaining for characterization of NSCs

For characterization of primary NSCs, the medium was removed completely, and primary neurospheres were fixed with 4% paraformaldehyde at room temperature for 30 min. After that, neurospheres were blocked with a blocking solution consisting of 0.2% bovine serum albumin (Santa Cruz Biotechnology, Dallas, TX), 0.1% Triton X100 (Wako Pure Chemicals, Osaka, Japan), and 0.1% NaN3 (Katayama Chemical, Osaka, Japan) at room temperature for 1 h and incubated with primary antibodies overnight at 4°C. Neurospheres were washed with PBS and incubated with secondary antibodies at room temperature for 2 h. The following antibodies were used: primary antibodies, Nestin (Abcam, Cambridge, UK, ab6142), SRY-box transcription factor 2 (SOX2) (Abcam, ab97959), glial fibrillary acidic protein (GFAP) (Abcam, ab7260), βIII tubulin (TUJ1) (Abcam, ab18207), Cluster of differentiation molecule 11b (CD11b) (Abcam, ab75476), secondary antibodies, Alexa Fluor 488 (Thermo Fisher Scientific) or Alexa Fluor 594 (Thermo Fisher Scientific). The nuclei were stained with Hoechst (Thermo Fisher Scientific) at room temperature for 15 min. Imaging was performed using a BZ-X800 microscope (Keyence).

#### Transcriptome analysis

Microarray analysis was performed as previously described (Ando et al., 2024; Yamazaki et al., 2024). Total RNA from E20.5 NSCs was analyzed using Clariom S Rat Arrays (Thermo Fisher Scientific). Differentially expressed genes (DEGs) were defined as those with FDR-corrected *p* < 0.05, absolute fold-change >2, and signal intensity >4. Gene Ontology (GO) analysis was performed using Metascape (Zhou et al., 2019). Gene set enrichment analysis (GSEA) was performed using GSEA v4.3.2 (Subramanian et al., 2005).

#### qPCR

Total RNA was isolated from the hippocampal tissues and NSCs using TRIzol reagent (Thermo Fisher Scientific). For mRNA expression analysis, total RNA was reverse transcribed into cDNA using M-MLV Reverse Transcriptase (Nippon Gene, Tokyo, Japan) with random hexamers as primers (TaKaRa, Otsu, Japan). qPCR was performed using the THUNDERBIRD Next SYBR qPCR Mix (Toyobo, Osaka, Japan) in the QuantStudio 7 Flex system (Thermo Fisher Scientific, MA, USA). The PCR primers used in this study were listed in Supporting Information (Table S4). The levels of target genes were normalized by using β-actin (*Actb*) as an internal control and relative gene expression was calculated using the 2^−ΔΔCt^ method.

#### Western blotting

Hippocampal tissues and NSCs were homogenized in RIPA buffer (Wako Pure Chemicals) with Protease Inhibitor Cocktails (Thermo Fisher Scientific), and the protein concentrations were measured using the Pierce BCA Protein Assay Kit (Thermo Fisher Scientific). Proteins were separated by SDS-PAGE and transferred onto PVDF membranes. The membranes were blocked with PVDF Blocking Reagent (Toyobo) at room temperature for 60 min. After blocking, primary antibodies were incubated overnight at 4°C, followed by incubation with the appropriate secondary antibodies at room temperature for 2 h. The following primary antibodies were used: β-actin (Abcam, ab49900), DNMT3A (Cell Signaling Technology, MA, USA, 3598), OPN (Santa Cruz, sc-21742). Chemiluminescent signals were detected using an Amersham ImageQuant 800 system (GE Healthcare, Chicago, IL, USA), and band intensities were quantified using a FUSION Chemiluminescence Imaging System (M&S Instruments, Osaka, Japan).

#### DNMT activity analysis

Nuclear proteins were extracted using EpiQuik Nuclea Cell Extraction Kit (Epigentek, NY, USA). Hippocampal NSCs were pooled from three offspring derived from the same dam, and thus the biological replicate n corresponds to the number of dams. DNMT activity was quantified using 5 μg of freshly prepared nuclear proteins from NSCs with the fluorometric EpiQuik DNMT Activity Assay Ultra Kit (Epigentek) according to the manufacturer’s instructions. In this assay, strip wells were coated with cytosine-rich DNA substrates. During the 2 h reaction, the DNMT enzyme from the nuclear extract samples transfers a methyl group from the methyl donor molecule AdoMet to cytosine, to methylate the DNA substrate. The methylated DNA were then recognized by anti 5-methylcytosine antibodies. The methylated DNA content proportional to enzyme activity was quantified fluorescently using 530 nm excitation and 590 nm emission readings from a microplate reader (Arvo; PerkinElmer, MA, USA).

#### Luciferase assay

Plasmid constructs were generated according to our previous reports (Munetsuna et al., 2021), the *Spp1* promoter prediction region (*Spp1* gene exon I upstream 1000bp or 1500bp) was inserted into the pGL4.10 [luc2] vector (Promega, WI, USA). We generated the PCR fragment of the Spp1 gene exon I upstream 1000bp or 1500bp region by using the following primers: forward (1000bp): 5′-GCTCGCTAGCCTCGATGCTGTTCACTGTACCAAC-3′, reverse forward (1500bp): 5′-GCTCGCTAGCCTCGATATTTAAAACAGAATTTTGTAGG-3′, reverse (1000bp and 1500bp): 5′-CCGGATTGCCAAGCTCGGACCTCCCAGAATTTAA-3′. Rat E20.5 hippocampal NSCs were cultured to determine *Spp1* promoter region. After cells had grown to 70–90% confluency, NSCs were transfected with pGL4.10 [luc2] vector inserted *Spp1* upstream region and the pGL4.74 [hRluc/TK] vector for internal transfection control using NEON electroporation system (1600 V, 10 ms, 3 pulses) (Thermo Fisher Scientific). After transfection, NSCs were seeded into 96-well plates and incubated for 48 h. Luciferase activity was measured using a luminometer (Arvo; PerkinElmer) and the Dual-Luciferase Reporter Assay System (Promega) in accordance with the manufacturer’s instructions.

#### Analysis of CpG methylation

CpG methylation was analyzed by bisulfite pyrosequencing as previously described (Ando et al., 2024; Kageyama et al., 2022; Yamazaki et al., 2018). Genomic DNA was extracted from hippocampal NSCs using NucleoSpin Tissue (TaKaRa, Otsu, Japan) and bisulfite-converted using the EpiTect Fast DNA Bisulfite Kit (Qiagen, Hilden, Germany). Bisulfite-modified DNA was amplified by PCR using methylation-specific primers designed with PyroMark Assay Design SW 2.0 (Qiagen) (Table S5). Quantitative methylation analysis was performed using a PyroMark Q24 Advanced system (Qiagen).

#### Small interfering RNAs (siRNA) experiments and transfection

siRNAs targeting *Dnmt3a* and *Spp1* were synthesized by Thermo Fisher Scientific. The following sequences were used: siDnmt3a, 5′-ACAAGGAAGUUUACACCGATT-3′ (sense), 5′-UCGGUGUAAACUUCCUUGUAA-3′ (antisense), siSpp1, 5′-GUAAGGAAGAUGAUAGGUATT-3′ (sense), 5′-UACCUAUCAUCUUCCUUACTC-3′ (antisense). Rat E20.5 hippocampal NSCs were incubated with siRNA or scramble RNA (Scr) at 37°C for 24 h. Cells were transfected with 30 nM of siRNAs using the Lipofectamine RNAiMAX reagent (Thermo Fisher Scientific).

#### sOPN treatment and iOPN transfection

To assess the effects of sOPN on NSC function, we used OPN recombinant protein (R&D Systems, MN, USA, 6359-OP-050). After seeding NSCs and preculture, neurosphere assay and neural differentiation induction were performed. All experiments were performed in OPN treated medium (0.02, 0.20, 1.00 μg/ml). Plasmid was generated to overexpress iOPN for assessment of its effects on NSC function. As previously described (Leavenworth et al., 2015; Shinohara et al., 2008), OPN sequences removed the signal peptide sites were cloned into pLL3.7 vector. We generated the PCR fragment of the iOPN by using the following primers: forward: 5′-AATTTAAATCGGATCATGCTCCCGGTGAAAGTG-3′, reverse: 5′-TCGCGGCCGCGGATCTTAATTGACCTCAGAAGATG-3′. The vector was introduced into the NSCs using NEON electroporation system (1600 V, 10 ms, 3 pulses) (Thermo Fisher Scientific), followed by drug selection with puromycin for 5 days. After selection, for assessment of NSC function, cells were seeded as required for each experiment. Plasmid-transfected cells expressed GFP; therefore, GFP^+^ neurospheres were used to assess proliferation, and TUJ1^+^ GFP^+^ cells were used to evaluate neural differentiation and morphology.

#### Statistical analysis

All data are expressed as means ± SD. Statistical analyses were performed using JMP v.14 (SAS Institute, NC, USA) or R version 4.5.2 (R Foundation for Statistical Computing, Vienna, Austria). Body weight and caloric intake were analyzed using one-way ANOVA followed by Bonferroni post hoc test, whereas other comparisons were performed using Student’s t test. Statistical significance was set at *p* < 0.05. Effect sizes for pairwise comparisons were calculated using Cohen’s d, and 95% confidence intervals were estimated. For multiple comparisons, *p* values were adjusted using the Holm–Bonferroni method. The experimental unit for all *in vivo* and *ex vivo* experiments was the individual animal. The number of dams (litters) and offspring is indicated in the figure legends; typically, 1–3 offspring per dam were included per group. For BrdU quantification, multiple sections per brain were analyzed and summed to generate a single value per animal. For NSC functional assays, multiple wells derived from the same animal were averaged to yield one value per animal. Offspring were derived from multiple independent litters; however, litter was not included as a factor in the statistical models. For *in vitro* experiments, the number of independent experiments is indicated in the figure legends.

## Supplemental information

Document S1. Figures S1–S7 and Tables S1–S5

Document S2. Article plus supplemental information
