# Supplementary material for: Neural stem cells as potential mediators of prenatal dietary stress through epigenetic mechanisms
Source: Stem Cell Reports. 2026 Jul 2;21(7):102996. doi: 10.1016/j.stemcr.2026.102996 (PMC13385421; doi:10.1016/j.stemcr.2026.102996)
Supplement: Document S1. Figures S1–S7 and Tables S1–S5 [file mmc1.pdf]

Stem Cell Reports, Volume *21*

Supplemental Information

Neural stem cells as potential mediators of prenatal dietary stress through epigenetic mechanisms

Itsuki Kageyama, Hiroya Yamada, Mirai Yamazaki, Takuya Wakasugi, Yuri Kamiya, Masaki Ohshiro, Manaka Ito, Yoshiki Tsuboi, Takashi Watanabe, Genki Mizuno, Yoshitaka Ando, Hiroaki Ishikawa, Koji Suzuki, Koji Ohashi, and Eiji Munetsuna

SUPPLEMENTAL FIGURES AND LEGENDS

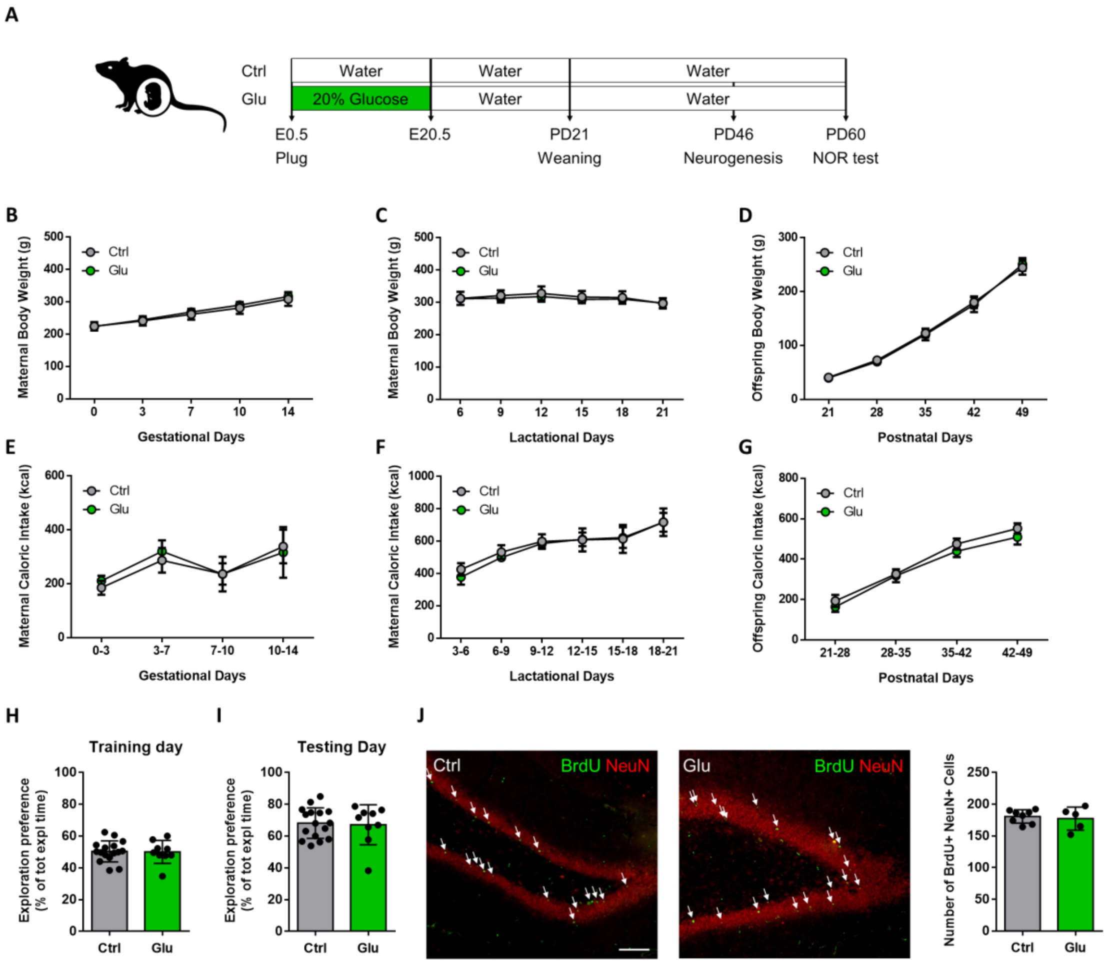

Figure S1. Maternal glucose intake does not impair hippocampus-dependent memory and adult neurogenesis in offspring.

(A) Animal models and experimental schedule for analysis of hippocampal function in offspring.

(B-D) Body weight of dams and offspring during the experimental period (dam: n = 4-22/group; offspring: n = 9-24/group).

(E-G) Caloric intake of dams and offspring during the experimental period. Caloric intake indicates the sum from diets and drinking water (dam: n = 4-22/group; offspring: n = 4-18/group).

(H and I) The percentage of exploration time spent on the novel object in the training (H) and testing day (I) (dam: n = 3-6/group; offspring: n = 9-16/group).

(J) Immunostaining for BrdU and NeuN in the hippocampal DG (dam: n = 3-4/group; offspring: n = 5-8/group). White markers indicate $BrdU^+$ $NeuN^+$ cells. Scale bar: 100 µm.

Ctrl, control group; Glu, Glucose group. n = number of animals analyzed. Values are presented as means ± SD. For (B-G), statistical analysis was performed by one-way ANOVA. For (H-J), statistical analysis was performed by Student's t test. Nonsignificant comparisons are not shown.

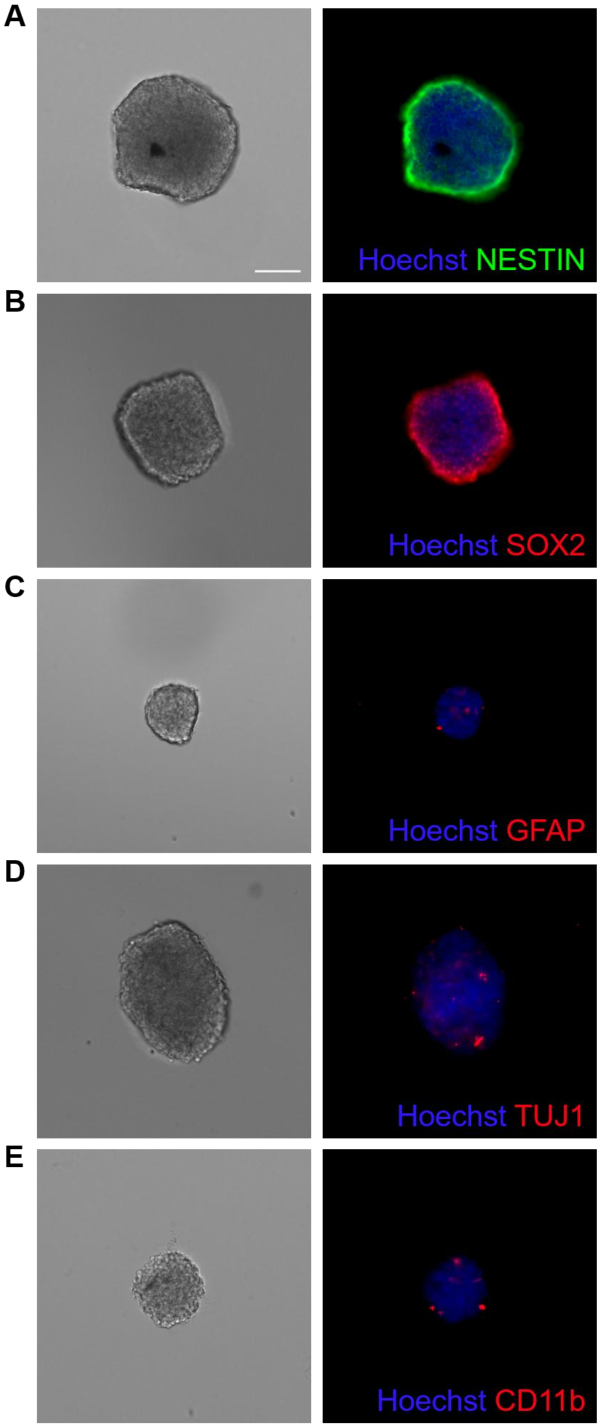

Figure S2. Immunocytochemical characterization of neural stem cells (NSCs).

(A) NESTIN, a neural stem cell marker. Scale bar: 100 μm.

(B) SOX2, a neural stem cell marker.

(C) GFAP, an astrocytic marker.

(D) TUJ1, a neuronal marker.

(E) CD11b, a microglial marker.

NESTIN and SOX2 were robustly expressed in neurospheres, whereas minimal signal for GFAP, TUJ1, and CD11b was detected, supporting the enrichment of NSCs in the cultured spheres.

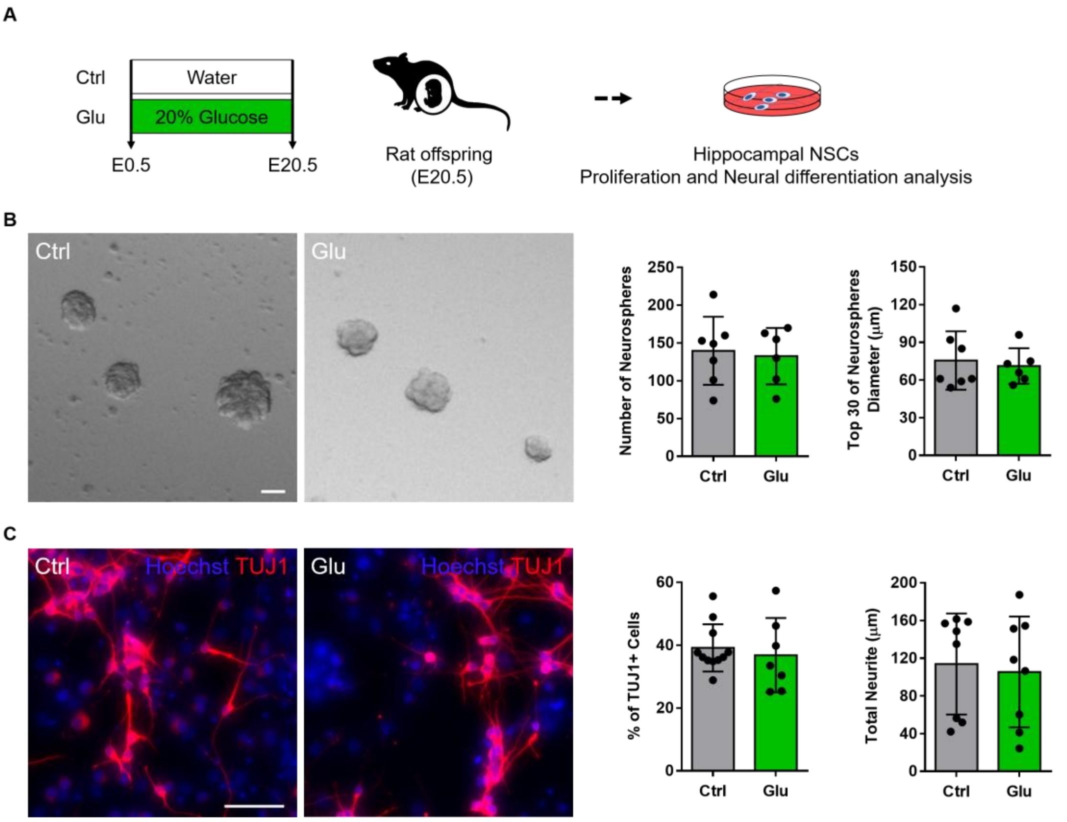

Figure S3. Maternal glucose intake does not decrease hippocampal NSC proliferation and neural differentiation in offspring at E20.5.

(A) Schematic of isolation and culture of NSCs from rat offspring in Glucose models.

(B) Neurosphere formation and quantification of neurosphere diameters at E20.5 (dam: n = 3-5/group; offspring: n = 6-7/group). Scale bar: 50 μm.

(C) Immunostaining for quantification of the percentage and total neurite length of TUJ1$^+$ cells differentiated from hippocampal NSCs at E20.5 (dam: n = 3-6/group; offspring: n = 7-11/group). Scale bar: 50 μm.

Ctrl, control group; Glu, Glucose group. n = number of animals analyzed. Values are presented as means ± SD. All statistical analysis was performed by Student's t test. Nonsignificant comparisons are not shown.

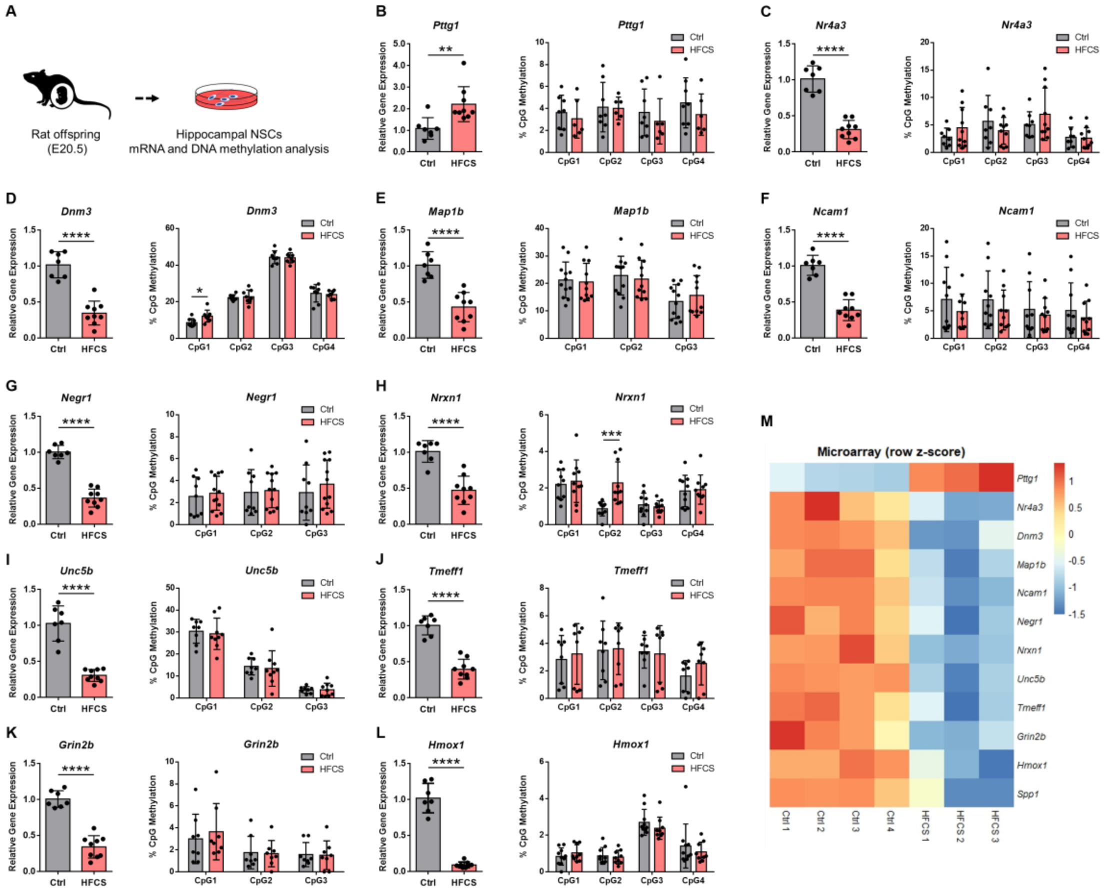

Figure S4. Gene expression and DNA methylation levels of genes associated with NSC function that showed changes by microarray analysis in E20.5 NSCs in HFCS group.

(A) Primary culture of hippocampal NSCs at E20.5.

(B-L) qPCR and pyrosequencing analysis showed mRNA expression and DNA methylation status of hippocampal NSCs at E20.5 (dam: n = 3-5/group; offspring: n = 6-12/group).

(M) Heatmap of microarray expression levels for the 12 selected genes, including *Spp1*, in hippocampal NSCs at E20.5 (dam: n = 2/group; offspring: n = 3–4/group).

Ctrl, control group; HFCS, HFCS group. n = number of animals analyzed. Values are presented as means ± SD. All statistical analysis was performed by Student's t test. Nonsignificant comparisons are not shown. $*p < 0.05$, $**p < 0.01$, $***p < 0.001$, $****p < 0.0001$.

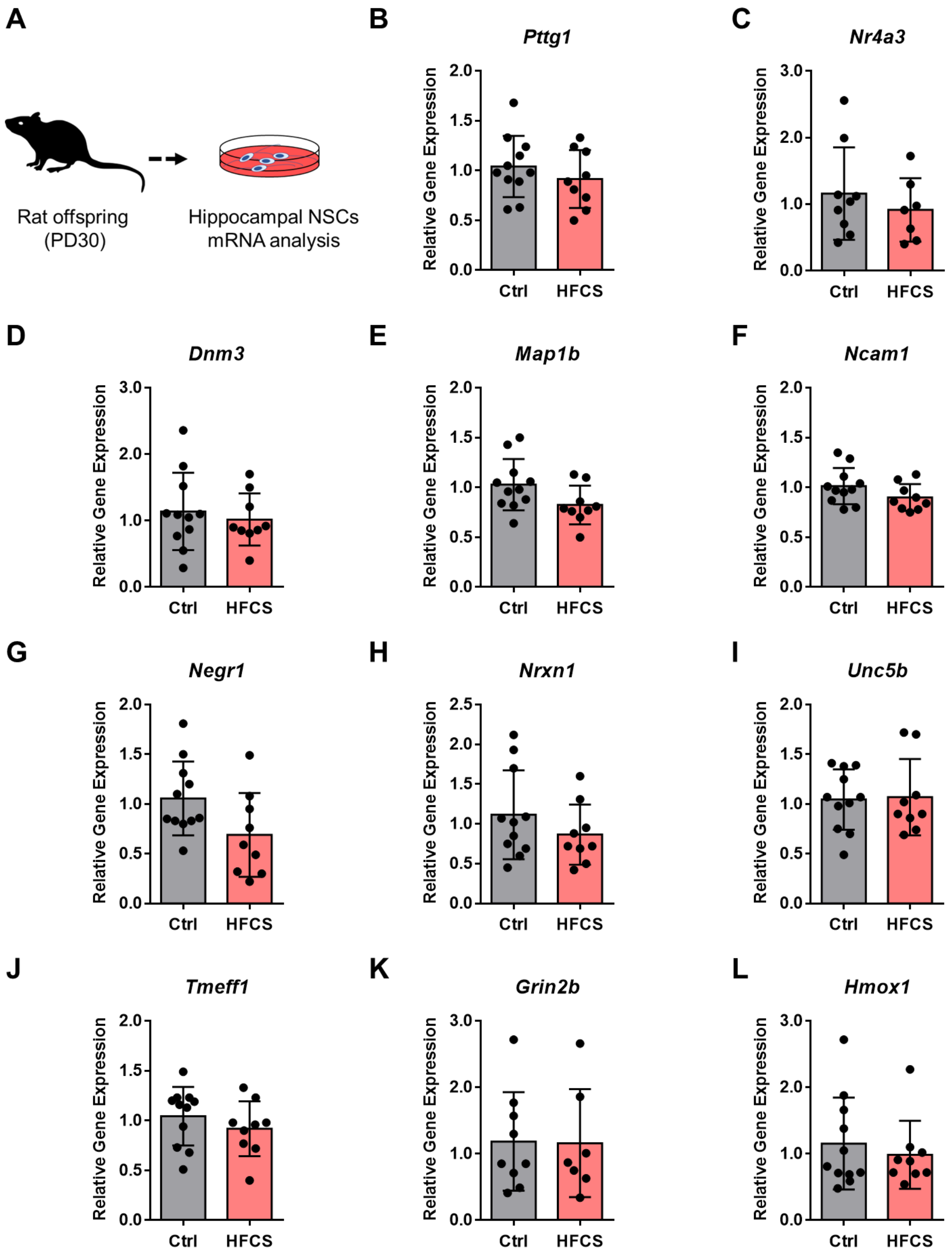

Figure S5. Gene expression of genes associated with NSC function that showed changes by microarray analysis in PD30 NSCs in HFCS group.

(A) Primary culture of hippocampal NSCs at PD30.

(B-L) qPCR analysis showed mRNA expression of hippocampal NSCs at PD30 (dam: n = 3-4/group; offspring: n = 7-11/group).

Ctrl, control group; HFCS, HFCS group. n = number of animals analyzed. Values are presented as means ± SD. All statistical analysis was performed by Student's t test. Nonsignificant comparisons are not shown.

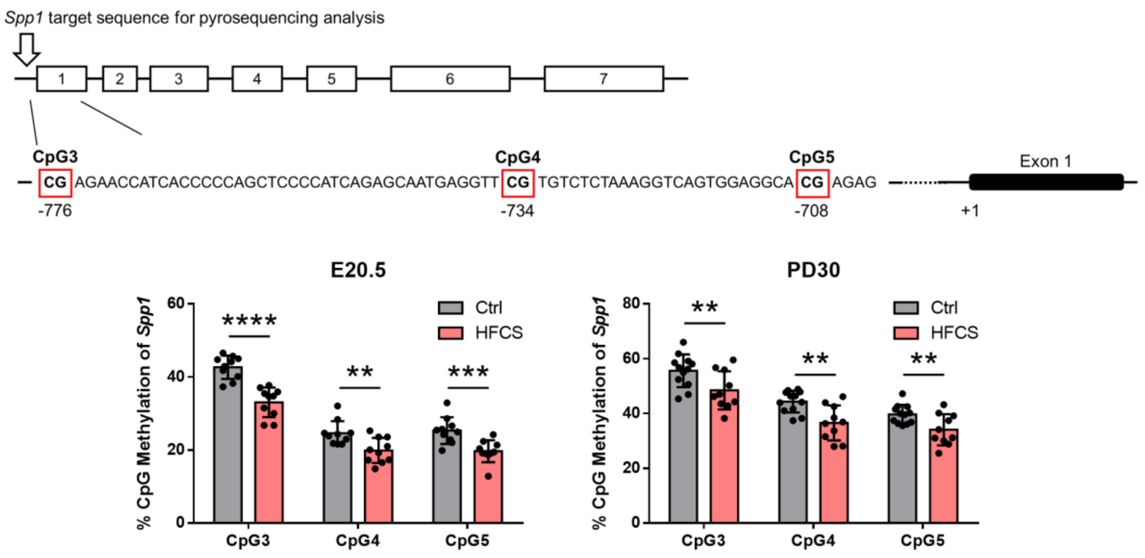

Figure S6. Excess maternal high-fructose corn syrup intake alters DNA methylation at multiple CpG sites in the *Spp1* promoter region of hippocampal NSCs in offspring at E20.5 and PD30.

The analyzed sequence of the *Spp1* promoter region is shown schematically. DNA methylation analysis of *Spp1* promoter region (CpG3, 4, 5) by pyrosequencing of hippocampal NSCs at E20.5 and PD30 (dam: n = 4-5/group; offspring: n = 10-12/group).

Ctrl, control group; HFCS, HFCS group. n = number of animals analyzed. Values are presented as means ± SD. All statistical analysis was performed by Student's t test. Nonsignificant comparisons are not shown. $^{**}p < 0.01$; $^{***}p < 0.001$; $^{****}p < 0.0001$.

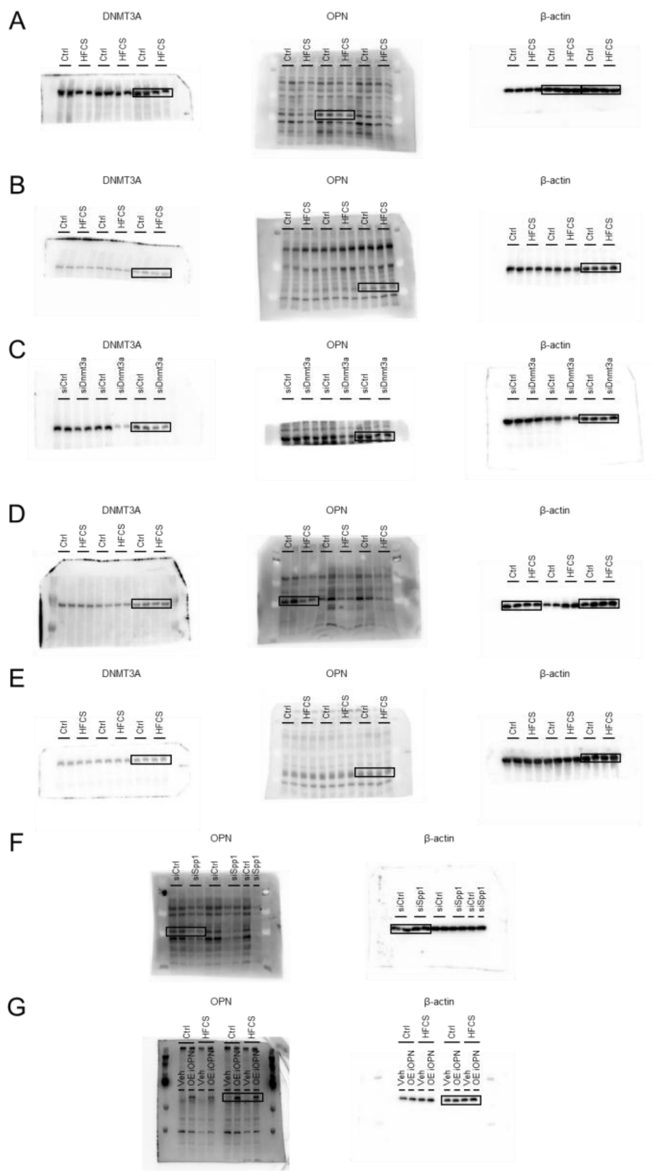

Figure S7. Full-length Western blot images corresponding to Figures 4–7.

(A) E20.5 NSCs in Figure 4C and 4F.

(B) E20.5 hippocampal tissue in Figure 4K.

(C) siDnmt3a-transfected NSCs in Figure 4N.

(D) PD30 NSCs in Figure 5C and 5F.

(E) PD30 hippocampal tissue in Figure 5I.

(F) siSpp1-transfected NSCs in Figure 6B.

(G) iOPN-overexpressing NSCs in Figure 7F.

Ctrl, control group; HFCS, HFCS group; siCtrl, siDnmt3a or siSpp1 control group; siDnmt3a, siDnmt3a group; siSpp1, siSpp1 group; Veh, Vehicle group; OE iOPN, Over Expression iOPN group. β-actin was used as a loading control. Target proteins and β-actin were detected on independent membranes. Quantification in the main figures was performed after normalization to β-actin. Bands shown in the main figures are indicated by boxes.

SUPPLEMENTAL TABLES

Table S1. Top 20 genes list of upregulated genes.

Gene Symbol	Ctrl Avg (log2)	HFCS Avg (log2)	Fold Change	Adjust p-val	FDR p-val
Hist1h2ail; *LOC682330*	8.99	14.17	36.41	6.55E-05	0.010
Ns5atp9	7.14	12.29	35.56	4.17E-05	0.009
Tk1	6.06	11.00	30.74	6.73E-05	0.010
Hist1h2bl; *Hist1h2bf*	7.45	12.32	29.23	4.16E-05	0.009
LOC690126	11.07	15.84	27.17	8.06E-05	0.011
Hist1h2ail1	6.68	11.34	25.42	9.17E-05	0.011
LOC102549061	9.48	13.87	21.00	4.48E-05	0.009
Aurkb	6.00	10.21	18.59	1.41E-05	0.007
Hist1h3c; *LOC102549173*	7.16	11.19	16.36	2.55E-05	0.008
Rab3b	9.24	12.97	13.25	2.60E-05	0.008
Tmem100	7.94	11.61	12.74	1.37E-05	0.007
Olfm3	10.74	14.29	11.71	3.82E-05	0.009
Hist1h2bd	8.88	12.42	11.67	9.59E-05	0.012
Kif22; *KIF22*	7.90	11.42	11.52	4.95E-05	0.009
Mcm3	6.89	10.37	11.20	1.21E-05	0.007
Rrm2	10.30	13.76	11.00	2.47E-05	0.008
Cdca3	8.29	11.71	10.74	8.98E-05	0.011
Fam64a	5.02	8.41	10.54	2.41E-05	0.008
Pbk	7.90	11.26	10.30	6.90E-06	0.006
Ccnb2	9.69	13.04	10.19	2.16E-05	0.008

Table S2. Top 20 genes list of downregulated genes.

Gene Symbol	Ctrl Avg (log2)	HFCS Avg (log2)	Fold Change	Adjust p-val	FDR p-val
Gdf15	14.48	6.66	-226.28	7.18E-05	0.010
Serping1	12.17	6.17	-63.85	1.13E-05	0.007
Cd68	11.54	6.18	-41.15	2.80E-05	0.008
Fcer1g	9.95	5.17	-27.61	8.76E-06	0.007
Spp1	8.84	4.86	-15.80	6.64E-05	0.010
Lyz2	10.70	7.03	-12.76	1.78E-05	0.008
Mpeg1	8.22	4.69	-11.60	1.70E-06	0.005
Fabp4	8.43	4.97	-11.03	3.71E-05	0.009
Hmox1	17.10	13.80	-9.84	1.19E-05	0.007
Laptm5	9.16	6.00	-8.94	6.18E-06	0.006
Cd4	9.00	5.85	-8.88	6.50E-07	0.003
Gad2	10.43	7.35	-8.47	7.29E-05	0.010
Lilrb4	8.66	5.83	-7.10	7.76E-07	0.003
Tmem130	12.33	9.63	-6.47	7.20E-05	0.010
Slc6a15	12.08	9.42	-6.31	2.63E-06	0.005
Gria1	10.31	7.65	-6.31	2.17E-05	0.008
Grin2b	10.63	8.01	-6.14	8.01E-05	0.011
Gadd45g	12.48	9.90	-5.97	6.98E-05	0.010
Slc2a3	11.72	9.17	-5.86	1.66E-05	0.008
Slc2a3; *LOC100909595*	11.72	9.17	-5.86	1.66E-05	0.008

Table S3. Representative DNA methyltransferase genes list identified by microarray.

Gene Symbol	Ctrl Avg (log2)	HFCS Avg (log2)	Fold Change	Adjust p-val	FDR p-val
Dnmt3a	10.21	9.55	-1.58	0.006	0.062
Dnmt3b	5.31	5.33	1.02	0.311	0.604
Dnmt1	9.12	9.81	1.60	0.076	0.279

Table S4. Primer sequence for qPCR.

Gene		Primer sequencing
Dnmt3a	Forward	5'-TGCCAGTCATCCGCCACCTC-3'
	Reverse	5'-CTCCGTCCTCTCGTTCTTGGTG-3'
Spp1	Forward	5'-CCAGCCAAGGACCAACTACA-3'
	Reverse	5'-AGTGTTTGCTGTAATGCGCC-3'
Pttg1	Forward	5'-GGCGTAGTCTTGGGTCTCTC-3'
	Reverse	5'-GTGGCGTTGAAACCTGCAAT-3'
Nr4a3	Forward	5'-GGAAACGTGGCGACATCCTA-3'
	Reverse	5'-GATTGGGGGAGAGTCACAGC-3'
Dnm3	Forward	5'-CTCCACAGGTTCCATCCAG-3'
	Reverse	5'-TGAGGACTCTAGTGGACGGATT-3'
Map1b	Forward	5'-TGCTTCTGCATCCAAGTCAG-3'
	Reverse	5'-CTTGGCCGTCTTAGTGGTTC-3'
Ncam1	Forward	5'-GTCTGCATCGCTGAGAACAA-3'
	Reverse	5'-AGTTCCATGGCTGTCTGATTC-3'
Negr1	Forward	5'-GCTTCTGAGCCTGTGCTCTT-3'
	Reverse	5'-CCGTCTTCCAAGTAACACCTG-3'
Nrxn1	Forward	5'-GGCAAATTGAGAGAGGATGC-3'
	Reverse	5'-CACACTCCTTGATTTGAACA-3'
Unc5b	Forward	5'-GGTCTACTGTCTGGAGGACACTC-3'
	Reverse	5'-CCAAGTAGCCACCCAGAGTC-3'
Tmeff1	Forward	5'-AAGTGTGGACCGTGCAAGTA-3'
	Reverse	5'-ACCCGCTGCAGTCTATGTTAC-3'
Grin2b	Forward	5'-ATGCAAGCGAGAAGAGGACC-3'
	Reverse	5'-AGCTAGTCGGCTCTCTTGGT-3'
Hmox1	Forward	5'-AGCCTGGTTCAAGATACTACC-3'
	Reverse	5'-GGGGCCAACACTGCATTTAC-3'
Actb	Forward	5'-ATTGGTGGCTCTATCCTGGC-3'
	Reverse	5'-CAGCTCAGTAACAGTCCGC-3'

Table S5. Primer sequence for DNA methylaton analysis.

Gene		Primer sequencing
Spp1	Forward	5'-AGGTTATATAGGGTATTGATTGTAGAA-3'
	Reverse	5'-CACACAATAACCCTAAATTCCTCTC-3'
	Sequencing (CpG1, 2)	5'-GGTAGTTTGATTTTTGTAGTT-3'
	Sequencing (CpG3)	5'-TTTATGTGGTTATTATAGTTTAGAT-3'
	Sequencing (CpG4, 5)	5'-AGTTTTTTATTAGAGTAATGAGG-3'
Pttg1	Forward	5'-AGGGGGAGGTGGTTGAAAAT-3'
	Reverse	5'-CCCCACCCTACTCCAACCTCCAAT-3'
	Sequencing	5'-AGTGTGTAGTTGTTAAAGATG-3'
Nr4a3	Forward	5'-TAGTGGTAGTGGAGGTGGGAAA-3'
	Reverse	5'-ACTTTTAAACAAATCCCCATATTAACT-3'
	Sequencing	5'-GGTAGTAGTAGGAGATTGGA-3'
Dnm3	Forward	5'-GGTTTTGGAGTTAGGGTGAAAG-3'
	Reverse	5'-CCCAAACTAAAAAACAAAATACTACACTT-3'
	Sequencing	5'-GTGTATGGTGAAGTTGAT-3'
Map1b	Forward	5'-GGTTTATGTTTTTGGGTGGAGTA-3'
	Reverse	5'-TTCCTTACCCCAAACCATCA-3'
	Sequencing	5'-GTGATTGTTGTAGTTATTTAA-3'
Ncam1	Forward	5'-GTTTGAGTGAAGGAAAAGGGTGAAAAGAA-3'
	Reverse	5'-AAAATAAATAACAACCCCAACCT-3'
	Sequencing	5'-GGAAGGATATAGTGAGG-3'
Negr1	Forward	5'-AGAGGTTAGTTGGTAGTTTTTTAATAG-3'
	Reverse	5'-AACCCCCCACTTTAAAATACTCAAATT-3'
	Sequencing	5'-TTAATTTATTTATAGTTTTG-3'
Nrxn1	Forward	5'-TTGGAAGGGGTTGTAGTTGTTAG-3'
	Reverse	5'-ACCAAACCCAAAAACATATATAATCAAAAC-3'
	Sequencing	5'-ATGGTATTGAGGAGAGTTTA-3'
Unc5b	Forward	5'-GGTGATAATGAAGGAGATAGTTAAGTAGTT-3'
	Reverse	5'-ATCAAACACCCAAATCCTACTAA-3'
	Sequencing	5'-ATTATATTTTAGGATGAGTTATTTT-3'
Tmeff1	Forward	5'-AAGGTTTATAAAGGGTTTTTGAGATATAG-3'
	Reverse	5'-CCCTCCCCAAAAACCATTATTCCT-3'
	Sequencing	5'-GTTTGATTAGTTAGAAGGATT-3'
Grin2b	Forward	5'-GTAGTTAGGGGGTGGGAAAA-3'
	Reverse	5'-TAACCACTATCTTACTCCCCTCAC-3'
	Sequencing	5'-GGGAAAAGTTTAATTTGAGT-3'
Hmox1	Forward	5'-TGGAGTTTGTAGGAGTAGAGTTA-3'
	Reverse	5'-CCTTAATCCCCCAACCTACATTTCTAAAC-3'
	Sequencing	5'-GTTTATAGTTAGATAGGTAA-3'
